# Rethinking the Current Older-people-first Policy for COVID-19 Vaccination in Japan

**DOI:** 10.2188/jea.JE20210263

**Published:** 2021-09-05

**Authors:** Kenji Matsui, Yusuke Inoue, Keiichiro Yamamoto

**Affiliations:** 1 Division of Bioethics and Healthcare Law, National Cancer Center, Tokyo, Japan; 2 Department of Public Policy, The Institute of Medical Science, The University of Tokyo, Tokyo, Japan; 3 Office of Bioethics, The Center for Clinical Sciences, The National Center for Global Health and Medicine, Tokyo, Japan

**Keywords:** COVID-19, vaccination prioritization policy, ethics

As of May 16, 2021, only 2.5% of Japan’s 36 million older people (aged ≥65 years) had received at least one dose of the coronavirus disease (COVID-19) vaccine.^1^ Even among the 4.8 million medical workers given first priority, the reported completion rate of the initial vaccination is 72.3%.^1^ Due to this slow implementation, Japan is currently ranked 120^th^ in vaccination rate among 206 countries/territories, the worst among the 37 Organisation for Economic Co-operation and Development member countries.^2^ These statistics imply that, at the current pace, most healthy younger people (aged 20–59 years), who account for exactly half of the entire population and form the core labor force in Japan (around 52 million), will need to wait until at least next year to be vaccinated.

Medical workers and patients with underlying diseases aside, the older-people-first policy for COVID-19 vaccination was developed through vigorous discussion within the Committee on Vaccination Basic Policy of the Inoculation/Vaccination Working Group of the Health Science Council at the Ministry of Health, Labour and Welfare. The main reason for this prioritization was that older people were considered more vulnerable; namely, they were at higher risk of developing serious complications and death than younger people. However, in view of the changes in social circumstances discussed below, this policy should be reconsidered.

First, Japan’s economy has shrunk more than expected due to the slow vaccine rollout and self-restraint in going out.^3^ Second, job-hunting younger people have experienced the biggest drop in job availability in 46 years.^4^ Third, there has been a significant increase in suicides, particularly among younger workers, since the beginning of the COVID-19 pandemic.^5^ Fourth, cases are surging among younger people due to the emergence of new variants, such as N501Y.^6^^,^^7^ Fifth, quite a few younger people, and younger workers in particular, have valid reasons for not being able to adhere to preventive practices, such as the need to commute to work.^8^ Sixth, Japan’s birth rate, and therefore the population of the next generation which will sustain the future Japanese society, has declined significantly due to COVID-19’s impact on the pregnancy rate and worsening economic conditions.^9^ Seventh, regardless of age, many have suffered from ‘cabin fever’ due to the prolonged restrictions, such as social requests to “stay at home,” for more than a year.^10^ Eighth, research using mathematical modeling from a Swedish group found that “the disease-induced herd immunity level may be substantially lower than the classical herd immunity level…a reduction…from 60% under homogeneous immunization down to 43%…in a structured population,” if we take account of age cohorts and social activity levels—namely, assuming that younger people tend to take more risks and more frequently maintain their pre-pandemic lifestyle, such as actively contacting others, than older people.^11^ If this model applies to Japan in its current state, only by vaccinating younger people can sufficient herd immunity be achieved.

Taken together, these factors indicate the need to rethink the older-people-first policy on vaccination prioritization in Japan, if the vaccines are safe and useful. The possibility of shifting from the current policy to an equal distribution policy which includes younger people should be explored. In fact, Indonesia prioritizes those of productive age (18–59 years) for vaccination more highly than older people.^12^ Our proposed equal-opportunity-for-vaccination policy, however, does not go as far as Indonesia’s. Rather, the policy must consider a healthy balance from an ethical standpoint as well. In this sense, our proposed policy can be ethically justified based on the ethical principle of equal opportunity or distributive justice.^13^ Our suggested change to the policy is to treat younger people as equally vulnerable cohorts as older people from public policy and economic perspectives.
